# Modifiable psychosocial factors associated with psychological distress, depression, anxiety and self-rated health among Aboriginal and Torres Strait Islander populations

**DOI:** 10.1017/S2045796026100584

**Published:** 2026-03-31

**Authors:** Subash Thapa, Julaine Allan, Santosh Giri, Hazel Dalton, Kedir Y. Ahmed, Jamie Newman, Peter Gibbs, Damien Little, Phillip Naden

**Affiliations:** 1 Rural Health Research Institute (RHRI), Charles Sturt University, Orange, NSW, Australia; 2 Discipline of General Practice, School of Medicine, Adelaide University, Adelaide, SA, Australia; 3 Orange Aboriginal Medical Service (OAMS), Orange, NSW, Australia; 4 Regional Enterprise Development Institute (REDI.E), Dubbo, NSW, Australia; 5 Coonamble Aboriginal Health Service (CAHS), Coonamble, NSW, Australia

**Keywords:** depression, Indigenous Australians, mental health, psychosocial wellbeing, social support

## Abstract

**Aims:**

To examine mediators and modifiable psychosocial factors associated with psychological distress, depression, anxiety and self-rated health among Aboriginal and Torres Strait Islander peoples (hereafter respectfully referred to as ‘Indigenous Australians’) aged ≥18 years.

**Methods:**

This was a cross-sectional study based on the analysis of the 2018–19 National Aboriginal and Torres Strait Islander Health Survey dataset (*N* = 3942). Odds ratios (OR) and 95% confidence intervals (CI) for associations and indirect effects for mediation analyses were computed.

**Results:**

Our results showed that Indigenous Australians with higher levels of perceived social support were less likely to have psychological distress (OR = 0.36, 95% CI: 0.23, 0.56), depression (OR = 0.44, 95% CI: 0.29, 0.67), anxiety (OR = 0.43, 95% CI: 0.28, 0.65) and low self-rated health (OR = 0.52, 95% CI: 0.33, 0.82). Similarly, those with a high level of mastery were less likely to have psychological distress (OR = 0.14, 95% CI: 0.11, 0.19), depression (OR = 0.20, 95% CI: 0.15, 0.28), anxiety (OR = 0.26, 95% CI: 0.20, 0.36), and low self-rated health (OR = 0.37, 95% CI: 0.28, 0.50). Perceived social support mediated 33.7% of the association between removal from the natural family and psychological distress, 14.6% of the association between discrimination and psychological distress, 20.3% of the association between discrimination and depression, 14.8% of the association between discrimination and anxiety and 16.6% of the association between discrimination and low self-rated health. Both perceived social support and mastery mediated the association between physical harm and psychological distress, depression and anxiety.

**Conclusions:**

We believe that community-driven psychosocial programs that enhance social support, self-efficacy and cultural connection may significantly improve the mental health and psychosocial well-being of Indigenous Australians.

## Background

Australian Indigenous populations face a disproportionately higher burden of mental disorders, accounting for 23% (equivalent to 55,200 years) of the total years of healthy life lost within these communities, compared to just 12% for the broader Australian population in 2022 (Australian Institute of Health Welfare, 2022). These mental health challenges are closely linked to lifestyle factors, chronic health conditions, suicide and premature mortality, contributing to 20% of the Indigenous health gap in Australia (Australian Institute of Health Welfare, 2022). The National Agreement on Closing the Gap aims to overcome the inequalities experienced by Indigenous Australians, achieve life outcomes equal to those of all Australians and reduce the suicide rate towards zero by the year 2031 (Holland, 2018; Department of the Prime Minister and Cabinet, 2024). However, there has been slow progress in achieving the targets, such as reducing incarceration rates, lowering youth detention rates, reducing suicide rates (age-standardized rate of 20.8 per 100,000 in 2021), and closing the life expectancy gap (Department of the Prime Minister and Cabinet, 2024).

Intergenerational trauma and ongoing socio-economic and cultural harms are well-established as drivers of the mental health burden and high suicide rates in Australian Indigenous communities (Markwick *et al.*, 2014; Black *et al.*, 2015; Kairuz *et al.*, 2021; Thapa *et al.*, 2023, 2024b). Addressing these challenges requires a comprehensive approach that focuses on social capital and fostering a sense of mastery over personal circumstances. Global research also highlights the importance of psychological well-being and social relationships in mitigating the effects of structural disadvantages, which act as stressors shaping mental health and well-being (Thoits, 1985; Daniel *et al.*, 2006; Veer *et al.*, 2021; Wang *et al.*, 2024). Social capital is known as a foundation for resilience and resource accessibility, while a strong sense of mastery enables individuals to effectively manage stress and adversity.

In the Australian context, Indigenous social and emotional well-being is distinct from Western mental health frameworks. Cultural knowledge, connection to Country, family and kinship and a strong social identity are essential contributors to mental health and well-being (Verbunt *et al.*, 2021; Thapa *et al.*, 2024a). While related constructs such as social capital or loneliness are also known to influence mental health (Diener *et al.*, 2008), social support and mastery offer distinct yet interrelated perspectives on psychosocial well-being, capturing both relational connectedness and individual agency (Tsai *et al.*, 2016; Fürtjes *et al.*, 2023). Social support reflects the sense of being cared for and having reliable networks (Tsai *et al.*, 2016), whereas mastery reflects self-efficacy in managing life challenges (Fürtjes *et al.*, 2023). These constructs are particularly relevant in Indigenous contexts because they are meaningful and aligned with local values and lived experiences, representing actionable targets for community-led interventions (Day and Francisco, 2013).

Despite their conceptual and practical relevance, no studies to date have specifically examined the impact of social support and mastery on mental health outcomes within Australian Indigenous communities. A critical public health question thus remains: to what extent can the harms associated with past adverse experiences on the mental health and well-being of Indigenous Australians be mitigated by addressing modifiable community-level psychosocial factors, such as perceived social support and mastery? Both constructs are measurable using validated instruments within large-scale datasets, including the 2018–19 National Aboriginal and Torres Strait Islander Health Survey (NATSIHS), allowing for population-level insights (Australian Bureau of Statistics, 2019). This combination of cultural relevance, modifiability, and measurability underscores the need to investigate these psychosocial factors, providing the rationale for the current study. Therefore, this study examined the mediators and modifiable psychosocial factors associated with psychological distress, depression, anxiety and self-rated health among Indigenous Australians aged 18 years and older.

## Methods

### Study design and data sources

This cross-sectional study examined the 2018–19 NATSIHS dataset. The 2018–19 NATSIHS was designed to collect a wide range of information about the health and wellbeing of Indigenous Australians, including individuals’ demographics, nutrition, social determinants of health, chronic diseases including mental health conditions and experiences of harm (Australian Bureau of Statistics, 2019). Funding for the survey was provided by the Australian Government Departments of Health and Prime Minister and Cabinet.

### Sampling procedures and sample size

The 2018–19 NATSIHS was conducted by the Australian Bureau of Statistics (ABS) between July 2018 and April 2019. The survey used different sampling strategies to select the study participants from community and non-community Indigenous populations. The community sample included a random selection of discrete Indigenous communities and associated outstations from the Dwelling Register for Aboriginal and Torres Strait Islander Communities.

**Figure 1.**
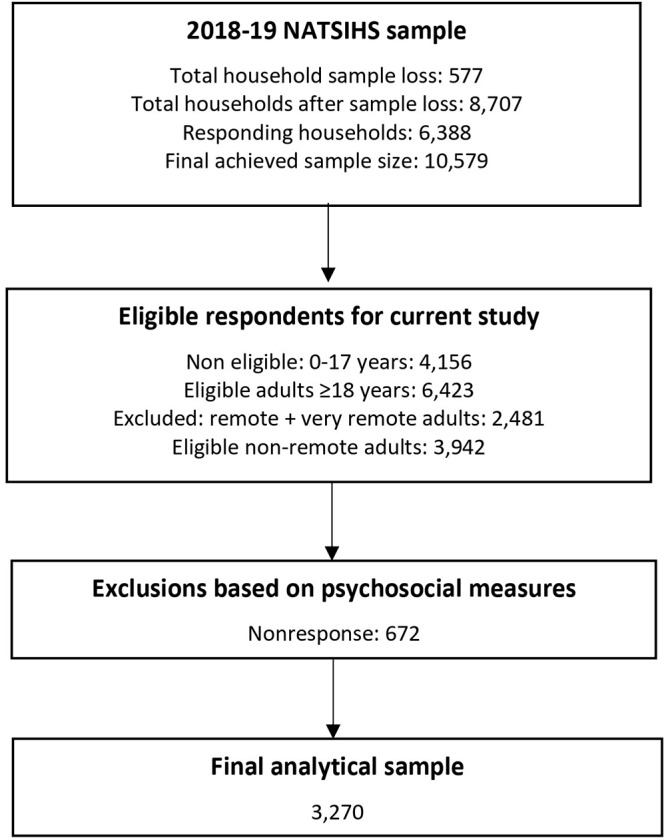
Flow diagram for the final analytical sample for the mental health component of the 2018–19 National Aboriginal and Torres Strait Islander Health Survey (NATSIHS).

The non-community sample involved multistage area sampling of private dwellings outside Indigenous communities. Mesh blocks with Indigenous households from the 2016 census were identified. Mesh blocks are the smallest geographical units defined in the Australian Statistical Geography Standard (ASGS), typically containing 30 to 60 dwellings. Dwellings in each mesh block were randomly selected, and up to two adults aged 18 years or older were randomly selected from both the community and non-community samples (Australian Bureau of Statistics, 2019). Only respondents from non-remote areas and those aged 18 years and over were included in the study, as psychological variables were only measured in this group of the study population (Fig. 1).

Of the 3942 eligible participants in the NATSIHS 2018–19 dataset, 672 participants (17%) were excluded from the analyses due to missing data on key variables, such as perceived social support, level of mastery and adverse experiences (Fig. 1).

### Outcome variables

The outcome variables were psychological distress, depression, anxiety and self-rated health. Psychological distress was measured using the Kessler-5 (K5), a modified version of the Kessler-10 designed specifically for use with Aboriginal and Torres Strait Islander people. The scores ranged from 0 to 25 (0–5: low or no distress, 6–11: moderate distress, 12–19: high distress and 20–25: very high distress) and were dichotomized into ‘high’ (>12) and ‘low’ (≤12). Depression and anxiety were identified at the time of the survey based on whether the respondent had been diagnosed with depression or anxiety disorder by a health professional and expected to persist for 6 months or more (Uher *et al.*, 2014; Australian Bureau of Statistics, 2019). Classification of mental health conditions in the NATSIHS adhered to the International Statistical Classification of Diseases and Related Health Problems, 10th Revision (ICD-10) (Australian Bureau of Statistics, 2019). Development and cultural adaptation of the tools to measure the outcome variables were informed through advice from an expert advisory panel, and the questions were tested in non-remote and remote areas of Australia. Self-assessed health was based on the single question: ‘How would you rate your current state of health?’, which was rated on a 5-point Likert scale (1 = High to 5 = Low) and was later dichotomised into ‘high’ (1–3) and ‘low’ (4–5).

### Explanatory variables

The explanatory variables were broadly categorised into two groups: psychosocial factors and adverse experiences in the past. The psychosocial factors included cultural identity, perceived social support and level of mastery. The adverse experiences included discrimination because of Aboriginal and Torres Strait Islander backgrounds (e.g., being called names, hearing racist comments, being ignored, not trusted, unfairly arrested, humiliated, having objects thrown at them), physical harm in the last 12 months, and ever removed from one’s natural family. Table 1 presents the definitions of all the variables used in the study.

**Table 1.** Definitions of study variables

Variables	Definition
**Psychosocial factors**	
Affiliation to an Indigenous or language group	Identify as being affiliated to a specific mob or language group. Grouped as ‘1’ = ‘yes’ or ‘2’ = ‘no’.
Perceived social support	The Multidimensional Scale of Perceived Social Support (MSPSS) is used to measure a person’s perception of the level of social support they receive from family and friends (Australian Bureau of Statistics, 2019). Respondents rated each item on a 7-point scale, ranging from 1 = very strongly disagree to 7 = very strongly agree. Social support contributes to resilience and can reduce the effect of adverse life-events (Dudgeon *et al.*, 2014). Like the Pearlin Mastery Scale, the MSPSS was only asked of people in non-remote areas. Grouped as ‘1’ = ‘Low’ (mean score 1.0−2.9), ‘2’ = ’Moderate’ (3.0−5.0), or ‘3’ = ’High’ (5.1−7.0).
Level of mastery	The Pearlin Mastery Scale measured the level of mastery felt by Indigenous Australians; that is, how much a person feels in control of life events and outcomes. The scale consisted of seven items, with each item rated on a 4-point agree–disagree scale. Scores for all items were summed to produce a total mastery score, with higher scores indicating greater perceived mastery. High levels of mastery are important because they can reduce the effect of stress on a person’s physical and mental wellbeing (Australian Bureau of Statistics, 2019). Like the MSPSS, the mastery questions were only asked of people in non-remote areas. Grouped as ‘1’ = ‘High’ (score 20−28) or ‘2’ = ’low’ (7−19).
**Past adverse experiences**	
Unfair treatment in the last 12 months	Defined as having experiences of unfair treatment (e.g., called names, racial comments, ignored, not trusted, unfairly arrested, humiliated, having objects thrown at them) because of Indigenous backgrounds in the last 12 months. Grouped as ‘1’ = ‘yes’ or ‘2’ = ‘no’.
Removed from natural family at some point	Defined as ever been removed from natural family by the government. Grouped as ‘1’ = ‘no’ or ‘2’ = ‘yes’.
Physical harm in the last 12 months	Defined as having experiences of physically hurt or harmed by someone on purpose, including physical fights in the last 12 months. Grouped as ‘1’ = ‘no’ or ‘2’ = ‘yes’.
**Socioeconomic factors**	
Personal income	The 2021 census median individual income (AUD 540 per week) for Indigenous Australians was used to dichotomise as ‘1’ = ‘below average’, ‘2’ = ‘average or above’.
Highest educational attainment	Grouped as ‘1’ = ‘Did not complete year 12’, ‘2’ = ‘Complete year 12’, ‘3’ = ‘Certificate/diploma’, or ‘4’’ = ‘Higher education’.
**Health risk factors**	
Daily smoking	Defined as smoking any number of cigarettes daily and categorised as ‘1’ = ‘non-smoker’, or ‘2’ = ‘non-daily or former smoker, or ‘3’ = ‘daily smoker’.
Excessive alcohol drinking	‘Excessive’ drinking was defined as consuming over 21 units (168 g or 213 ml) of alcohol weekly, based on criteria outlined in a recent publication in Lancet Public Health and guidelines from the National Health and Medical Research Council (NHMRC) (National Health and Medical Research Council, 2020; See *et al.*, 2023). Grouped as ‘1’ = ‘non-excessive’ or ‘2’ = ‘excessive drinking’
Physical activity	Defined according to the 2014 Physical Activity and Sedentary Behaviour Guidelines (Department of Health, 2021). Participants were categorised based on whether they met the recommended weekly activity levels ≥ 150 minutes of moderate-intensity activity (or equivalent combination of moderate- and vigorous-intensity activity) per week. Categorised as ‘1’ = ‘active’ (met the guideline) or ‘2’ = ‘inactive’ (did not meet the guideline).
Central obesity	Central obesity defined as a waist circumference of ≥ 102 cm for males and ≥ 88 cm for females.(Australian Department of Health and Aged Care, 2021) Grouped as ‘1’ = ‘no’ or ‘2’ = ’yes’.

### Potential covariates

Informed by previous research (Thapa *et al.*, 2024a), we selected the covariates, which included socioeconomic factors (personal income and highest educational attainment), health risk factors (daily smoking, excessive alcohol consumption, physical inactivity and central obesity) and demographic characteristics (age, sex, marital status).

### Statistical analysis

All data were accessed in the ABS DataLab for analysis. Frequencies and percentages were initially calculated to present an overview of the study population. All frequencies and percentages were weighted using the personal weight variable (fingerwt), and the sample size was approximated by dividing the personal weight variable by 100. Multilevel logistic regression models were used to test the association between explanatory variables and outcomes. Odds ratios (ORs) with 95% confidence intervals (CIs) were computed.

To investigate whether the effect of past adverse experiences on mental health outcomes is mediated by perceived social support and level of mastery, we performed mediation analysis using a counterfactual approach (Lange *et al.*, 2012; VanderWeele, 2015). We fitted natural effect models using an imputation-based approach to assess the total causal effect (TCE) of unfair treatment, physical harm and removal on mental health outcomes (adjusted for covariates), and to decompose the association into the natural direct effect (NDE) and the natural indirect effect (NIE) through perceived social support and level of mastery (Vansteelandt *et al.*, 2012). The indirect effects represent the portion of the total effects of unfair treatment, physical harm and removal mediated by perceived social support and level of mastery, while the direct effects represent the remaining of the total effect. The TCE is the sum of the NDE and NIE (VanderWeele, 2015).

We hypothesised that the association between unfair treatment, physical harm and removal from family on mental health outcomes is partially mediated by perceived social support and level of mastery. We used generalised linear models (GLM) with logit link function to specify exposure variables and adjust for covariates. The mediation analyses were conducted in R programming (R version 4.4.1) (R. Core Team, 2024) using the package “medflex” (Steen *et al.*, 2017), and all remaining statistical analyses were conducted using Stata version 18.0 (StataCorp, USA) with ‘svy’ command to adjust for effect of sampling and stratification.

### Role of the funding source

Funders had no role in study design, data collection, data analysis, interpretation or writing of the report.

## Results

### Study participants

This study included a weighted sample of 3942 individuals, with a mean age of 37.3 years (SD = ± 17.8), and 52.0% (2039) were female. Of the total participants, 53.6% (2359) made more than the national average income, 60.4% (2290) completed Year 12, and 37.7% (1479) were married. About 37.3% (1465) smoked daily, 13.0% (509) drank alcohol at higher than recommended levels, 88.4% (3,467) were physically inactive and 57.6% (2,260) had central obesity.

A total of 9.2% (350) had perception of low social support, 34.4% (1300) had low mastery and 8.9% (351) knew that they were affiliated with a cultural or language group. A total of 26.3% (957) participants had experienced discrimination in the last 12 months and 5.9% (227) had experienced physical harm in the last 12 months. A total of 15.2% (571) of the participants were removed from their natural families by the government at some point (Table 2).

**Table 2.** Characteristics of study participants (*N* = 3942)

		Psychological distress		Depression		Anxiety		Self-rated health	
	Total	High	Low	Yes	No	Yes	No	Low	High
	*n* (%)	*n* (%)	*n* (%)	*n* (%)	*n* (%)	*n* (%)	*n* (%)	*n* (%)	*n* (%)
**Sex**									
Male	1884 (48.0)	496 (27.4)	1312 (72.6)	336 (16.0)	1760 (84.0)	334 (16.0)	1762 (84.0)	484 (23.1)	1612 (76.9)
Female	2039 (52.0)	735 (36.5)	1278 (63.5)	563 (25.1)	1679 (74.9)	701 (31.3)	1541 (68.7)	560 (26.8)	1642 (73.2)
**Personal income**									
Below national average	1822 (46.4)	760 (43.6)	984 (56.4)	580 (29.3)	1399 (70.7)	590 (29.8)	1390 (70.2)	675 (34.1)	1304 (65.9)
National average or above	2359 (53.6)	470 (22.7)	1606 (77.3)	319 (13.5)	2040 (86.5)	446 (18.9)	1913 (81.1)	409 (17.3)	1950 (82.7)
**Educational attainment**									
Did not complete Year 12	1501 (39.6)	504 (35.3)	926 (64.7)	327 (21.8)	1174 (78.2)	371 (24.7)	1130 (75.3)	472 (31.4)	1029 (68.5)
Completed Year 12	514 (13.5)	174 (35.0)	325 (65.0)	95 (18.6)	418 (81.4)	116 (22.6)	397)77.4)	80 (15.6)	433 (84.4)
Certificate/diploma	1446 (38.2)	432 (30.2)	999 (69.8)	344 (23.8)	1102 (76.2)	372 (25.7)	1074 (74.3)	356 (24.6)	1090 (75.4)
Higher education	330 (8.7)	71 (21.6)	258 (78.4)	46 (14.1)	283 (85.9)	77 (23.5)	252 (76.5)	75 (22.9)	254 (77.1)
**Marital status**									
Married	1479 (37.7)	362 (24.9)	1092 (75.1)	280 (18.9)	1205 (81.1)	336 (22.6)	1149 (77.4)	373 (25.1)	1112 (74.9)
Single	2444 (62.3)	869 (36.7)	1497 (63.3)	619 (21.7)	2234 (78.3)	699 (24.5)	2154 (75.5)	711 (24.9)	2142 (75.1)
**Smoking**									
Non-smoker	1356 (34.6)	321 (24.4)	993 (75.6)	235 (17.3)	1122 (82.7)	303 (22.4)	1053 (77.6)	260 (19.1)	1097 (80.9)
Nondaily or former smoker	1102 (28.1)	330 (30.9)	737 (69.1)	485 (17.1)	217 (19.7)	275 (25.0)	827 (75.0)	308 (27.9)	794 (72.1)
Daily smoker	1465 (37.3)	579 (40.3)	860 (59.7)	414 (27.6)	1058 (72.2)	401 (27.3)	1064 (72.7)	466 (31.8)	999 (68.2)
**Alcohol consumption**									
Not excessive	3414 (87.0)	1072 (32.1)	2264 (67.9)	787 (20.6)	3033 (79.4)	930 (24.4)	2890 (75.7)	948 (24.8)	2872 (75.2)
Excessive	509 (13.0)	159 (32.8)	325 (67.2)	111 (21.6)	406 (78.4)	105 (20.4)	412 (79.7)	136 (26.3)	381 (73.8)
**Physical inactivity**									
Inactive	3467 (88.4)	1145 (34.0)	2224 (66.0)	787 (22.7)	2680 (77.3)	895 (25.8)	2572 (74.2)	978 (28.2)	2489 (71.8)
Active	454 (11.6)	85 (19.0)	364 (81.0)	111 (13.2)	733 (86.8)	139 (16.5)	705 (83.5)	106 (12.5)	739 (87.5)
**Central obesity**									
No	1663 (42.4)	482 (29.8)	1132 (70.2)	300 (18.1)	1362 (81.9)	343 (20.6)	1320 (79.4)	278 (16.7)	1386 (83.3)
Yes	2260 (57.6)	749 (33.9)	1458 (66.1)	558 (24.7)	1702 (75.3)	636 (28.1)	1624 (71.9)	755 (33.4)	1506 (66.6)
**Perceived social support**									
Low	350 (9.2)	194 (56.0)	153 (44.0)	149 (42.6)	200 (57.4)	157 (44.9)	193 (55.2)	144 (41.2)	206 (58.8)
Moderate	1175 (30.8)	516 (44.3)	647 (55.7)	333 (28.4)	842 (71.6)	359 (30.6)	816 (69.4)	420 (35.7)	755 (64.3)
High	2291 (60.0)	505 (22.3)	1763 (77.7)	356 (15.6)	1935 (84.4)	438 (19.1)	1853 (80.9)	420 (18.3)	1871 (81.7)
**Level of mastery**									
High	2475 (65.6)	400 (16.2)	2070 (83.8)	266 (10.8)	2209 (89.3)	382 (15.4)	2093 (84.6)	545 (41.9)	755 (58.1)
Low	1300 (34.4)	799 (61.7)	496 (38.3)	561 (43.1)	739 (56.9)	558 (42.9)	742 (57.1)	426 (17.2)	2049 (82.8)
**Affiliation to a specific group**									
Yes	351 (8.9)	108 (31.1)	238 (68.9)	61 (17.5)	289 (82.5)	78 (22.2)	273 (77.8)	106 (30.3)	245 (69.8)
No	3572 (91.1)	1123 (32.3)	2352 (67.7)	838 (21.0)	3150 (79.0)	958 (24.0)	3030 (76.0)	978 (24.5)	3009 (75.5)
**Unfair treatment in the last 12 months**									
No	2687 (73.7)	745 (28.0)	1917 (72.0)	540 (19.3)	2257 (80.7)	610 (21.8)	2187 (78.2)	630 (22.5)	2167 (77.5)
Yes	957 (26.3)	421 (45.2)	511 (54.8)	294 (29.6)	700 (70.4)	326 (32.8)	668 (67.2)	342 (34.4)	652 (65.6)
**Physical harm in the last 12 months**									
No	3597 (94.1)	1089 (30.6)	2467 (69.4)	757 (20.3)	2982 (79.7)	870 (23.3)	2869 (76.7)	932 (24.9)	2807 (75.1)
Yes	227 (5.9)	112 (52.5)	102 (47.6)	86 (35.5)	156 (64.5)	92 (38.1)	150 (61.9)	74 (30.8)	168 (69.2)
**Removed from natural family**									
No	3173 (84.8)	970 (30.8)	2182 (69.2)	660 (20.8)	2513 (79.2)	751 (23.7)	2422 (76.3)	780 (24.6)	2394 (75.4)
Yes	571 (15.2)	242 (43.1)	319 (57.0)	173 (30.3)	398 (69.8)	183 (32.1)	388 (67.9)	197 (34.4)	374 (65.6)

**Figure 2.**
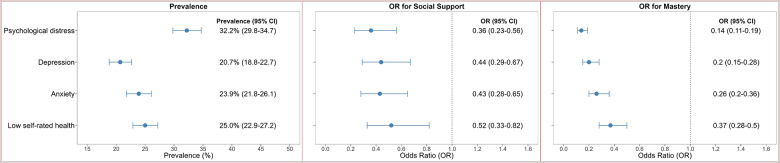
Prevalence and psychosocial determinants of psychological distress, depression, anxiety and low self-rated health among Indigenous Australians aged ≥18 years.

The prevalence of high psychological distress among Individuals aged 18 years or more was 32.2% (95% CI: 29.8, 34.7), with the rate 1.3 times higher among females (36.5%, 95% CI: 33.3, 39.9) than males (27.4%, 95% CI: 23.9, 31.2). The prevalence of depression was 20.7% (95% CI: 18.8, 22.7), with the rate 1.5 times higher among females (25.1%, 95% CI: 22.4, 28.1) than males (16.0%, 95% CI: 13.6, 18.7). Similarly, the prevalence of anxiety was 23.9% (95% CI: 21.8, 26.1), with the rate 2.0 times higher among females (31.3%, 95% CI: 28.2, 34.5) than males (15.9%, 95% CI: 13.4, 18.9). The prevalence of low self-rated health was 25.0% (95% CI: 22.9, 27.2), with the rate 1.2 times higher among females (26.7%, 95% CI: 24.0, 29.7) than males (23.1%, 95% CI: 20.1, 26.3) (Table 2, Fig. 2).

### Factors associated with psychological distress

Indigenous Australians with high levels of social support were less likely to experience psychological distress (OR = 0.36, 95% CI: 0.23, 0.56) compared to those with low social support. Those with high mastery had lower odds of psychological distress (OR = 0.14, 95% CI: 0.11, 0.19) compared with those with low mastery. Those who had experienced discrimination in the past 12 months were more likely to experience high psychological distress (OR = 2.24, 95% CI: 1.66, 3.03) than those who had not (Table 3).

**Table 3.** Logistic regression analysis for the association between psychosocial variables and psychological distress, depression, anxiety and low self-rated health among Indigenous Australians aged ≥ 18 years (2018/19) (*N* = 3270)

	Psychological distress		Depression		Anxiety		Low self-rated health	
	OR (95% CI)	*P*-value	OR (95% CI)	*P*-value	OR (95% CI)	*P*-value	OR (95% CI)	*P*-value
**Personal income**								
Above	1.00		1.00		1.00		1.00	
Below national average	1.85 (1.37,2.50)	<0.001	2.34 (1.72,3.19)	<0.001	1.70 (1.26,2.29)	<0.001	1.79 (1.33,2.42)	<0.001
**Educational attainment**								
Did not complete Year 12	1.00		1.00		1.00		1.00	
Completed Year 12	2.12 (1.34,3.37)	0.001	1.55 (0.95,2.52)	0.079	1.21 (0.76,1.93)	0.420	0.91 (0.55,1.50)	0.708
Certificate/diploma	1.32 (0.95,1.83)	0.096	1.80 (1.28,2.53)	0.001	1.31 (0.95,1.80)	0.091	1.06 (0.77,1.46)	0.717
Higher education	0.80 (0.44,1.47)	0.478	1.19 (0.66,2.15)	0.566	1.26 (0.74,2.15)	0.396	1.19 (0.69,2.06)	0.536
**Daily smoking**								
Non-smoker	1.00		1.00		1.00		1.00	
Nondaily or former smoker	1.60 (1.08,2.36)	0.021	1.06 (0.72,1.56)	0.765	1.38 (0.94,2.03)	0.098	1.18 (0.82,1.71)	0.382
Daily smoker	1.71 (1.23,2.39)	0.002	1.44 (1.02,2.06)	0.039	1.15 (0.82,1.62)	0.419	1.51 (1.04,2.19)	0.031
**Excessive drinking**								
No	1.00		1.00		1.00		1.00	
Yes	1.10 (0.70,1.79)	0.668	1.15 (0.72,1.84)	0.560	1.10 (0.70,1.73)	0.681	1.20 (0.75,1.92)	0.447
**Physical activity**								
Active	1.00		1.00		1.00		1.00	
Inactive	1.34 (0.86,2.08)	0.154	0.97 (0.60,1.57)	0.885	1.08 (0.70,1.69)	0.725	2.04 (1.30,3.22)	0.002
**Central obesity**								
No	1.00		1.00		1.00		1.00	
Yes	1.16 (0.85,1.58)	0.419	1.29 (0.95,1.75)	0.097	1.46 (1.08,1.97)	0.030	1.91 (1.39,2.63)	0.001
**Perceived social support**								
Low	1.00		1.00		1.00		1.00	
Moderate	0.70 (0.44,1.11)	0.182	0.61 (0.40,0.94)	0.024	0.61 (0.40,0.95)	0.030	0.85 (0.54,1.34)	0.547
High	0.36 (0.23,0.56)	<0.001	0.44 (0.29,0.67)	<0.001	0.43 (0.28,0.65)	<0.001	0.52 (0.33,0.82)	<0.001
**Level of mastery**								
Low	1.00		1.00		1.00		1.00	
High	0.14 (0.11,0.19)	<0.001	0.20 (0.15,0.28)	<0.001	0.26 (0.20,0.36)	<0.001	0.37 (0.28,0.50)	<0.001
**Affiliation to a specific group**								
Yes	1.00		1.00		1.00		1.00	
No	1.02 (0.62,1.67)	0.698	1.54 (0.98,2.41)	0.060	1.23 (0.78,1.94)	0.377	0.88 (0.52,1.44)	0.571
**Unfair treatment in the last 12 months**								
No	1.00		1.00		1.00		1.00	
Yes	2.24 (1.66,3.03)	<0.001	1.46 (1.09,1.96)	0.017	1.54 (1.15,2.05)	0.004	1.89 (1.38,2.59)	<0.001
**Physical harm in the last 12 months**								
No	1.00		1.00		1.00		1.00	
Yes	1.41 (0.79,2.52)	0.284	1.29 (0.80,2.08)	0.252	1.56 (0.90,2.69)	0.114	0.98 (0.57,1.60)	0.981
**Ever removed from the natural family**								
No	1.00		1.00		1.00		1.00	
Yes	1.37 (0.94,2.00)	0.117	1.38 (0.97,1.98)	0.076	1.33 (0.94,1.90)	0.110	1.11 (0.77,1.59)	0.579

OR: Odds ratio; CI: Confidence interval. Models adjusted for age, sex and marital status.

**Figure 3.**
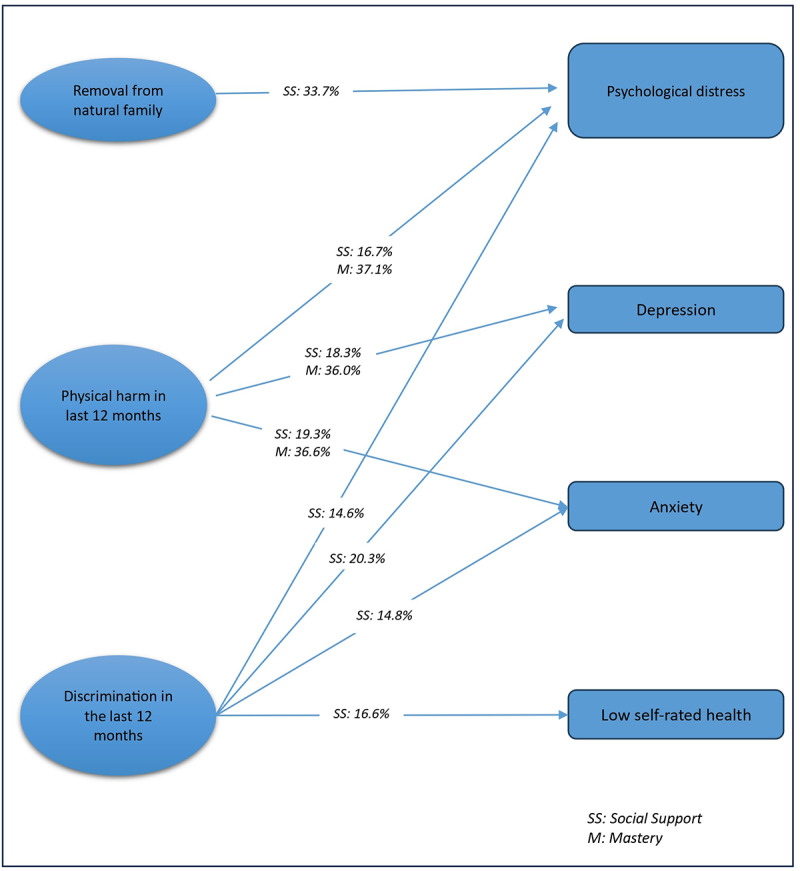
Mediation of perceived social support and level of mastery in the association between past adverse events (exposure) and mental health outcomes (outcome) among Indigenous Australians aged ≥18 years [Perceived social support and level of mastery act as mediators, and arrows indicate the indirect effects of past adverse events on mental health outcomes via social support and mastery].

The mediation analysis found that perceived social support mediated 33.7% of the association between a history of removal from the natural family and psychological distress (OR = 1.03, 95% CI: 1.02, 1.04) and 14.6% of the association between experiencing discrimination in the last 12 months and psychological distress (OR = 1.02, 95% CI: 1.02, 1.03). Perceived social support (OR = 1.04, 95% CI: 1.03, 1.06) and high levels of mastery (OR = 1.10, 95% CI: 1.07, 1.13) mediated 16.7% and 37.1% of the association between experiencing physical harm in the last 12 months and psychological distress, respectively (Table 4, Fig. 3).

**Table 4.** Adjusted direct and indirect associations of psychosocial variables and psychological distress, depression, anxiety and low self-rated health among Indigenous Australians aged ≥ 18 years (2018/19) (*N* = 3270)

	TCE	NDE	NIE	% NDE	% NIE
	OR (95% CI)	OR (95% CI)	OR (95% CI)	% (95% CI)	% (95% CI)
**Psychological distress**					
Social support mediation					
Unfair treatment	1.18 (1.14, 1.23)	1.15 (1.11, 1.20)	1.02 (1.02, 1.03)	85.4 (81.6, 87.9)	14.6 (12, 16.3)
Physical harm	1.3 (1.21, 1.39)	1.24 (1.16, 1.33)	1.04 (1.03, 1.05)	83.3 (77.3, 86.9)	16.7 (14.4, 18)
Removed from natural family	1.09 (1.05, 1.14)	1.06 (1.02, 1.10)	1.03 (1.02, 1.04)	66.3 (38.5, 76.5)	33.7 (30.9, 41.2)
Mastery mediation					
Physical harm	1.3 (1.21, 1.39)	1.18 (1.10, 1.25)	1.10 (1.07, 1.13)	62.9 (51.7, 69.5)	37.1 (35, 38.4)
**Depression**					
Social support mediation					
Unfair treatment	1.10 (1.06, 1.14)	1.08 (1.04, 1.12)	1.02 (1.01, 1.03)	79.7 (69.1, 84.7)	20.3 (19.5, 20.7)
Physical harm	1.22 (1.14, 1.30)	1.17 (1.10, 1.25)	1.04 (1.02, 1.05)	81.7 (72.9, 86.1)	18.3 (16.6, 19.2)
Mastery mediation					
Physical harm	1.21 (1.14, 1.29)	1.13 (1.07, 1.20)	1.07 (1.05, 1.10)	64.0 (19.5, 71.4)	36.0 (35.8, 36.4)
**Anxiety**					
Social support mediation					
Unfair treatment	1.11 (1.08, 1.15)	1.10 (1.05, 1.13)	1.02 (1.01, 1.02)	85.2 (78.8, 88.5)	14.8 (12.5, 15.9)
Physical harm	1.18 (1.11, 1.26)	1.14 (1.07, 1.22)	1.03 (1.02, 1.05)	80.7 (68.8, 85.9)	19.3 (18.8, 19.6)
Mastery mediation					
Physical harm	1.18 (1.11, 1.26)	1.14 (1.04, 1.18)	1.06 (1.04, 1.08)	63.4 (42.5, 72.8)	36.6 (35.1, 40.0)
**Low self-rated health**					
Social support mediation					
Unfair treatment	1.10 (1.06, 1.14)	1.08 (1.04, 1.12)	1.02 (1.01, 1.12)	83.4 (73.7, 87.8)	16.6 (14.9, 17.4)

TCE: Total causal effect; NDE: Natural direct effect; NIE: Natural indirect effect. Models adjusted for all covariates.

### Factors associated with depression

Indigenous Australians with high levels of social support were less likely to be depressed (OR = 0.44, 95% CI: 0.29, 0.67) compared to those with low social support. Those with high mastery were less likely to be depressed (OR = 0.20, 95% CI: 0.15, 0.28) compared to those with low mastery. Those who experienced discrimination within the last year were more likely to be depressed (OR = 1.46, 95% CI: 1.09, 1.96) than those who did not (Table 3).

The mediation analysis showed that perceived social support mediated 20.3% of the association between experiencing discrimination and depression (OR = 1.02, 95% CI: 1.01, 1.03). Perceived social support (OR = 1.04, 95% CI: 1.02, 1.05) and the level of mastery (OR = 1.07, 95% CI: 1.05, 1.10) mediated 18.3% and 36.0% of the association between experiencing physical harm in the last 12 months and depression, respectively (Table 4, Fig. 3).

### Factors associated with anxiety

Indigenous Australians with high levels of social support were less likely to have anxiety compared to those with low levels of social support (OR = 0.43, 95% CI: 0.28, 0.65). Those with high mastery were less likely to have anxiety compared to those with low mastery (OR = 0.26, 95% CI: 0.20, 0.36). Those who had experienced discrimination in the past year were more likely to have anxiety compared to those who did not (OR = 1.54, 95% CI: 1.15, 2.05) (Table 3).

The mediation analysis showed that perceived social support mediated 14.8% of the association between experiencing discrimination in the last 12 months and anxiety (OR = 1.01, 95% CI: 1.02, 1.02). Perceived social support (OR = 1.03, 95% CI: 1.02, 1.05) and the level of mastery (OR = 1.06, 95% CI: 1.04, 1.08) mediated 19.3% and 36.6% of the association between experiencing physical harm in the last 12 months and anxiety, respectively (Table 4, Fig. 3).

### Factors associated with low self-rated health

Indigenous Australians with high levels of social support were less likely to have low self-rated health compared to those with low levels of social support (OR = 0.52, 95% CI: 0.33, 0.82). Those with high mastery were less likely to have low self-rated health compared to those with low mastery (OR = 0.37, 95% CI: 0.28, 0.50). Those who experienced discrimination in the last year were more likely to report low self-rated health compared to those who did not (OR = 1.89, 95% CI: 1.38, 2.59) (Table 3).

The mediation analysis showed that perceived social support mediated 16.6% of the association between experiencing discrimination in the last 12 months and low self-rated health (OR = 1.02, 95% CI: 1.01, 1.02) (Table 4, Fig. 3).

## Discussion

This study found that Indigenous Australians with high levels of perceived social support and a strong sense of mastery were less likely to experience psychological distress, depression, anxiety and low self-rated health. Perceived social support plays a crucial role in mitigating the mental health effects of the removal from natural family and discrimination. In contrast, both social support and mastery mediate the effects of physical harm on psychological distress, depression and anxiety.

Contributing to ongoing scholarly work on strength-based approaches to Indigenous health, our study provides the empirical evidence that, among other factors, perceived social support and mastery are critical psychosocial elements in mitigating the effects of past adverse experiences and high levels of social disadvantage on mental health among Aboriginal and Torres Strait Islanders. Particularly, enhancing social support could be a viable strategy for improving mental health and wellbeing in Indigenous communities. This highlights the potential of targeted interventions that strengthen social support networks, foster resilience, and deepen connections to Indigenous identity as public health strategies to alleviate mental health burdens (Gupta *et al.*, 2020).

Some of the successful targeted programs include cultural resilience initiatives, such as ceremonies, storytelling and arts, which foster a sense of belonging and self-worth; community support networks, including peer support groups and community yarning circles; and efforts to support Indigenous leadership and initiatives (McKenzie *et al.*, 2016; Upton *et al.*, 2021; O’Doherty *et al.*, 2023). For people diagnosed with mental illnesses, integrating traditional healing practices with contemporary mental health services and incorporating culturally adapted psychotherapy (cognitive behavioural therapy) can enhance treatment effectiveness. Increasing funding for these mental healthcare services and emphasising Indigenous leadership in mental healthcare delivery could help bridge the mental health gap and improve well-being among these communities (Kowanko *et al.*, 2009; Farah Nasir *et al.*, 2021; Kennedy *et al.*, 2022; Topp *et al.*, 2022).

Community-led initiatives, including Aboriginal Community Controlled Health Services (Jamieson *et al.*, 2025) and Elders-led programs (Busija *et al.*, 2018), provide important spaces for connection, identity, support and capacity building. For example, the Deadly Choices program in South East Queensland demonstrates how community-led initiatives can foster both social support and mastery in practice (Malseed *et al.*, 2014). This health promotion initiative combined health education, tobacco cessation support, sporting and cultural activities, leadership development and community-based healthcare services. By engaging participants in collective and skill-building activities, the program not only enhanced health literacy but also strengthened social support networks and participants confidence and capacity to manage both physical and mental health, promoting overall wellbeing in Indigenous communities (Malseed *et al.*, 2014).

Evidence from related evaluations indicates that similar, culturally grounded, Indigenous-led services can strengthen social connectedness, empowerment and self-determination (Haswell *et al.*, 2010; Dudgeon *et al.*, 2014; Dingwall *et al.*, 2015; Santiago *et al.*, 2022; Thapa *et al.*, 2024a). However, most of these initiatives have not been systematically evaluated for their effects on constructs such as social support or mastery. This highlights both the promise of these initiatives and the need for further research on their psychosocial outcomes. Systematic evaluation could identify the most effective components of Indigenous-led programs, facilitating their scale-up across states and nationally, while ensuring cultural relevance and community ownership through engagement with local leaders and stakeholders (Thapa *et al.*, 2024b).

Within the socio-ecological framework, our findings emphasise the critical role of the microsystem factors, such as engaging family, peers and community members through culturally grounded, relational approaches to mitigate the enduring effects of adverse experiences (Day and Francisco, 2013). In this view, integrating psychosocial initiatives that strengthen cultural identity, social connections and personal agency is necessary to advance Australia’s Closing the Gap agenda. Nonetheless, addressing broader structural factors remains essential for achieving and sustaining long-term positive impact on the health and wellbeing of Indigenous Australians.

### Limitations

This study also had limitations. First, the use of cross-sectional data presents difficulties in establishing a temporal relationship between explanatory variables and the outcome variable. For example, it is important to note that depression and anxiety themselves may reduce perceived social support and mastery, as prior studies have shown that symptoms of depression can lead to social withdrawal and diminished perceptions of available support, while anxiety can undermine feelings of control and self-efficacy (Burns *et al.*, 2011; Santini *et al.*, 2015). This also suggests that the associations observed may be bidirectional, and that social support and mastery could be both protective factors and outcomes of mental health status (Lakey and Cronin, 2008; Burns *et al.*, 2011).

Second, a limitation of using moderation analysis in cross-sectional data is the inability to establish temporal or causal relationships between the moderator, independent and dependent variables. Our results from moderation analyses should be interpreted cautiously, with an understanding that they may not reflect actual causal effects. Future longitudinal research is needed to disentangle these relationships. Third, most of the explanatory variables were measured based on self-reported questionnaires, which could be a source of recall bias.

Fourth, psychological measures, such as perceived social support and level of mastery, were only administered to non-remote Indigenous populations aged ≥18 years, which may introduce selection bias by excluding remote participants. Remote communities often experience different social, economic, and health contexts, including variations in access to health services, social support networks and exposure to stressors (Thapa *et al.*, 2023). Consequently, the relationships observed in this study may not fully generalize to remote populations, and associations could differ in magnitude or direction.

Fifth, non-response, survey weighting and missing data may have influenced representativeness. For instance, approximately 17% of participants were excluded due to missing data, primarily from the psychological measures, which may have introduced selection bias. Participants from remote areas, younger adults, or those with incomplete survey responses were underrepresented in the analytical sample, which could affect the generalizability of the findings to the broader Indigenous population. While prior research suggests broadly similar patterns of social determinants and mental health across Indigenous populations nationally (Thapa *et al.*, 2024a; Astawesegn *et al.*, 2025), yet caution is warranted when extrapolating these results to remote communities. Sixth, dietary and nutrition-related variables were not included, as the NATSIHS 2018–19 did not collect comprehensive nutrition data, limiting our ability to examine their potential influence on mental health. Lastly, some people might not have disclosed information about physical harm and unfair treatment to an interviewer due to a lack of privacy during the interview process.

## Conclusion

Our study found that, in the context of past adverse experiences such as family separation, physical harm and discrimination, high perceived social support and a strong sense of mastery have protective effects against psychological distress, depression, anxiety and low self-rated health. Both social support and mastery mediate the reduction in the association between historical and contemporary trauma and these mental health outcomes. Psychosocial interventions that strengthen cultural identity, social connections and individuals’ sense of control could enhance the psychosocial and emotional well-being, as well as the mental health of Aboriginal and Torres Strait Islander populations.

## Data Availability

Weighted prevalence estimates from the Australian Bureau of Statistics (ABS) surveys used in the current study are available on the ABS website. Aggregated weighted data from these surveys are also hosted by the ABS on their TableBuilder online platform, where users can perform limited analyses (e.g., calculating prevalence by age groups). The ABS also stores the deidentified unit record-level data that underly the national surveys on their DataLab online platform. Users can perform more detailed analyses, such as generating variables not available publicly, and all results are vetted by ABS staff before release. Access to ABS TableBuilder and the ABS DataLab requires formal approval processes.

## List of abbreviations

ABS: Australian Bureau of Statistics
ASGS: Australian Statistical Geography Standard
CI: Confidence Interval
ICD-10: International Statistical Classification of Diseases and Related Health Problems, 10th Revision
K5: Kessler-5
NATSIHS: National Aboriginal and Torres Strait Islander Health Survey
NDE: Natural direct effect (NDE)
NIE: Natural indirect effect
OR: Odds Ratio
TCE: Total causal effect
